# Cardiotoxicity After Anthracycline Treatment in Survivors of Adult Cancers: Monitoring by USCOM, Echocardiography and Serum Biomarkers

**DOI:** 10.4021/wjon635w

**Published:** 2013-03-06

**Authors:** Alessandro Pastore, Sandra Geiger, Dorothee Baur, Andreas Hausmann, Johanna Tischer, Sophia Horster, Hans Joachim Stemmler

**Affiliations:** a Med. Dept. III, Ludwig-Maximilians University of Munich, Campus Grosshadern, Munich, Germany; b Med. Dept. II, Ludwig-Maximilians University of Munich, Campus Grosshadern, Munich, Germany

**Keywords:** Cardiotoxicity, Anthracyclines, CW-Doppler, USCOM, Ultrasound

## Abstract

**Background:**

Anthracyclines are agents with a well known documented anti-tumoral activity. Cardiac side effects are the principal toxicity. Here we evaluate and monitor the onset of late anthracycline-induced cardiotoxicity with real-time CW-Doppler ultrasound cardiac output monitoring (USCOM®) and echocardiography in combination with serum biomarkers.

**Methods:**

Fifty-two patients without cardiac disease who had received an anthracycline-based regimen for various cancer types were included in this study. Patients’ hemodynamic parameters as stroke volume (SV USCOM (mL)) and ejection fraction (EF ECHOCARDIOGRAPHY (%)) were measured with USCOM and echocardiography and correlated to serum biomarkers (NT-pro-BNP and cTnT).

**Results:**

Eighteen patients (34.6%) developed cardiac disease (NYHA I-III). An increasing cumulative anthracycline dose was associated with a decrease of the EF determined by echocardiography as well the SV by USCOM and with a higher NYHA class. Those patients who experienced cardiac disease showed a reduction of the EF and SV and increased serum biomarkers.

**Conclusions:**

Real-time CW-Doppler USCOM, is a fast and reliable method to monitor late hemodynamic changes as a symptom of anthracycline-induced cardiotoxicity comparable to the findings by echocardiography and serum biomarkers.

## Introduction

Anthracyclines are high effective cytostatic with a broad spectrum of activity in various cancer types. The anti-tumor effect of anthracyclines based on DNA binding, production of free radicals and of causing irreversible DNA damage by inhibition of topoisomerase II [1, 2]. Cardiac damage by anthracyclines can not be attributed to their anti-cancer mechanisms. The heart is a post-mitotic organ, and the single mechanism of adaptation and repair is hypertrophy of the remaining myocardium [3, 4]. Anthracyclines are an integral component of therapy to common tumor types such as, breast carcinoma, gastric carcinoma, soft tissue sarcoma, small cell lung cancer, as well as lymphatic and hematological malignancies [5]. The successful use of anthracyclines is limited by the development and onset of cardiac complications, which currently represent the most feared, limiting toxicities of this class of anti-neoplastic agents [5]. Anthracycline-induced cardiotoxicity can distinguish an early, a late acute, and a chronic form. Due to the prolonged survival and significantly improved long-term forecasts in oncology, is gaining increasing importance in the chronic cardiotoxicity [5, 6].

Published data on the incidence of anthracycline-induced cardiotoxicity are heterogeneous and lie between 2.2% and 26% [7]. International Guidelines in Oncology define cardiotoxicity as an absolute decrease in left ventricular ejection fraction (LVEF) > 10% units, when the ejection fraction (EF) falls below the normal value of 50%, or an absolute EF decrease by > 20%, or a decline in LVEF > 5% if the initial EF was already below 50% [6].

The most specific method to diagnose myocardial changes during treatment with anthracyclines is endomyocardial biopsy [8]. But non-invasive methods of cardiac monitoring were much more established in clinical routine and consisted of radionuclideventriculography and echocardiography. The latter is recommended from the ACC (American College of Cardiology), the AHA (American Heart Association) and the ASE (American Society of Echocardiography) for cardiac monitoring of patients receiving anthracyclines [9, 10].

In general, the prognosis of anthracycline-induced cardiomyopathy is described as relatively poor [11]. In contrast to acute cardiotoxicity, the chronic form presented as largely irreversible heart failure as a result of recurrent damage to the cardiomyocytes themselves and their consecutive decline. The correlation between cumulative anthracycline dose and late cardiac toxicity is well documented [12-14].

ANP and BNP are important serological parameters for diagnosis, prognosis and response to treatment in patients with acute or chronic heart failure [15-19]. ANP and BNP are cleaved from the C-terminal end of their pro-hormones pro-BNP/pro-ANP and are released into the circulation together with the correspondent n-terminal fragment (N-terminal pro-BNP/NT-pro-BNP/N-terminal pro-ANP/NT-pro-ANP). In heart failure, ANP and BNP are released related to the ventricular filling pressure. The plasma concentrations increase in clinical symptomatic or asymptomatic systolic or diastolic dysfunction [20].

Cardiac troponins are proteins with three subunits known as troponin T (cTnT), troponin I (cTnI) and troponin C. Cardinale et al showed that elevated levels of troponin during treatment with chemotherapy are associated with a decrease of left ventricular ejection fraction (EF). Contrary, patients with no elevation of troponin levels during chemotherapy may show a transient reduction of left ventricular EF which returns to baseline in the long-term follow up [21-24], cTnI is a specific and sensitive marker for myocardial damage and can be suggestive for the extent of left ventricular dysfunction in an early stage of therapy [25, 26].

USCOM (USCOM Pty Ltd, Sydney, Australia) is a non-invasive hemodynamic monitoring system based on continuous-wave (CW) Doppler-principle with a transportable touch-screen monitor and 2.2 MHz ultrasound probe. Knobloch and Co-workers have shown that combining real-time CW-Doppler ultrasound and serum biomarkers is feasible to monitor the hemodynamic changes to cardiotoxic agents like the anthracyclines or trastuzumab [27, 28].

Aim of the study was to evaluate late cardiotoxicity with real-time CW-Doppler ultrasound cardiac output monitoring (USCOM), and echocardiography in combination with serum biomarkers in patients undergoing an anthracyclines based chemotherapy for at least 24 months.

## Patients and Methods

The present study was carried out in accordance with the Declaration of Helsinki of the World Medical Association and was approved by the Ethics Committee of the Medical Faculty of the Ludwig-Maximilians University in Munich. Informed consent was given by the patient prior to enter the study. The primary objective was to quantify late cardiotoxicity after anthracycline-based chemotherapy as a reduction of the stroke volume (SV) by USCOM, as well as the EF by echocardiography. Secondary objectives were the determination of myocardial markers (NT-pro-BNP and cTnT), taking into account the time interval between treatment and the cumulative dose and time of investigation.

### Patient selection

Patients who had received for at least 24 months an anthracycline-based regimen for hematologic malignancies or a solid tumor, a Karnofsky Perfomance Status of ≥ 70% and an age between 18 and 65 years were eligible. Cardiac EF determined by echocardiography had to be normal (EF ≥ 50%) prior to enter the study. Patients who had a positive history of coronary heart disease with cardiac dysfunction (> NYHA I), or an impaired left ventricular EF (LVEF) were not eligible.

### NYHA class

The New York Heart Association (NYHA) Functional Classification provides a simple way of classifying the extent of heart failure. Patients are categorized accordig to limitation of activities of daily living. The limitations/symptoms are in regards to normal breathing and varying degrees in shortness of breath and or angina pain. Class I: Cardiac disease, but no symptoms and no limitation in ordinary physical activity, for example, shortness of breath when walking, climbing stairs etc.; Class II: Mild symptoms (mild shortness of breath and/or angina) and slight limitation during ordinary activity; Class III: Marked limitation in activity due to symptoms, even during less-than-ordinary activity, for example, walking short distances (20 - 100 m). Comfortable only at rest; Class IV: Severe limitations. Experiences symptoms even while at rest. Mostly bed bound patients.

### USCOM

USCOM is a noninvasive, continuous wave (CW) Doppler technology based device for the direct real-time measurement of cardiac output. The device calculates the velocity time integral (VTI), stroke volume (SV) and heart rate (HR), cardiac output (CO). Measurement was performed with the patient in the horizontal or sitting position. All measurements were performed by the same investigator. Extensive validation studies against invasive thermodilution (PAC, Pulmonry Artery Catheter; PiCCO, Pulse induced Contour Cardiac Output) were performed in intensive ward settings, demonstrating high correlation and limits of agreement according to Bland-Altman analysis [29-31].

### Chemical laboratory test parameters

As relevant cardiac markers hs-TnT (high-sensitivity cardial troponin T) and NT-pro-BNP levels were determined on the same day of cardiac monitoring.

### Cumulative anthracycline dose

The anthracycline equivalent dose was obtained multiplying the specific cumulative dose for a specific index value. Index for Doxorubicin, Epirubicin, Daunorubicin, Mitoxantrone was 1, 0.67, 0.83 and 4, respectively [13, 32, 33].

### Statistical methods

The analysis was performed using descriptive statistical methods. Results are plot using Microsoft® Excel®.

## Results

### Patient characteristics

Between April 2008 and May 2009, fifty-two patients were enrolled. Median patients’ age was 60 years. Patients suffered from various cancer-types including 24 non-Hodgkin lymphomas (46%), 15 breast cancers (28.8%), 6 Hodgkin lymphomas (11.5%), 4 soft-tissue sarcomas (7.7%), 2 acute myeloid leukaemia’s (3.9%), and 1 chronic lymphatic leukaemia (2%). Two patients died a few weeks after the examination due to tumor progression. The median elapsed time after receiving an anthracycline-based regimen was 59 months (range: 25 - 123 months). Baseline characteristics are given in detail in Table 1.

**Table 1:** Baseline patients characteristics

		Median	N (n = 52)	%
Age (years)		60.1		
Karnofsky Performance Status (%)		90.1		
Gender				
Female			29	55.80%
Male			23	44.20%
Tumor Type				
Breast-Cancer			15	28.80%
Hodgkin Lymphoma			6	11.50%
NHL			24	46.10%
Soft tissue sarcoma			4	7.70%
CLL			1	2.00%
AML			2	3.90%
Elapsed time after anthracyclines		59 months	52	100%
25 - 36 mo			14	31.8%
37 - 60 mo.			9	20.5%
61 - 84 mo.			9	20.5%
85 - 108 mo.			7	15.8%
> 108 mo.			5	11.4%
NYHA Class after therapy				
0			34	65.4%
I-II			12	23.1%
II			4	7.7%
II-III			2	3.8%
NT-pro-BNP (pg/mL) and hs-TnT (pg/mL) after therapy	NT-pro-BNP median	hs-TnT median		
NYHA class 0	101.9	6.3	18	34.6%
NYHA class I-II	148.3	6.5	12	66.7%
NYHA class II	2047.3	42.7	4	22.2%
NYHY class II-III	1137.2	10.9	2	11.1%
Cumulative anthracycline dose *		Median ± SD		
Overall (mg/m^2^)		300 ± 113.27		
Doxorubicin (index 1.0)		300 ± 81.42	38	73.1%
Epirubicin (equivalent × 0.67)		368 ± 130.28	11	21.1%
Daunorubicin (equivalent × 0.83)		300 ± 0	2	3.8%
Mitoxantrone (equivalent × 4.0)		120 ± NA	1	1.9%

* The anthracycline equivalent dose was obtained multiplying the specific cumulative dose for an index. Index for Doxorubicin, Epirubicin, Daunorubicin, Mitoxantrone are 1, 0.67, 0.83 and 4, respectively.

**Figure 1:**
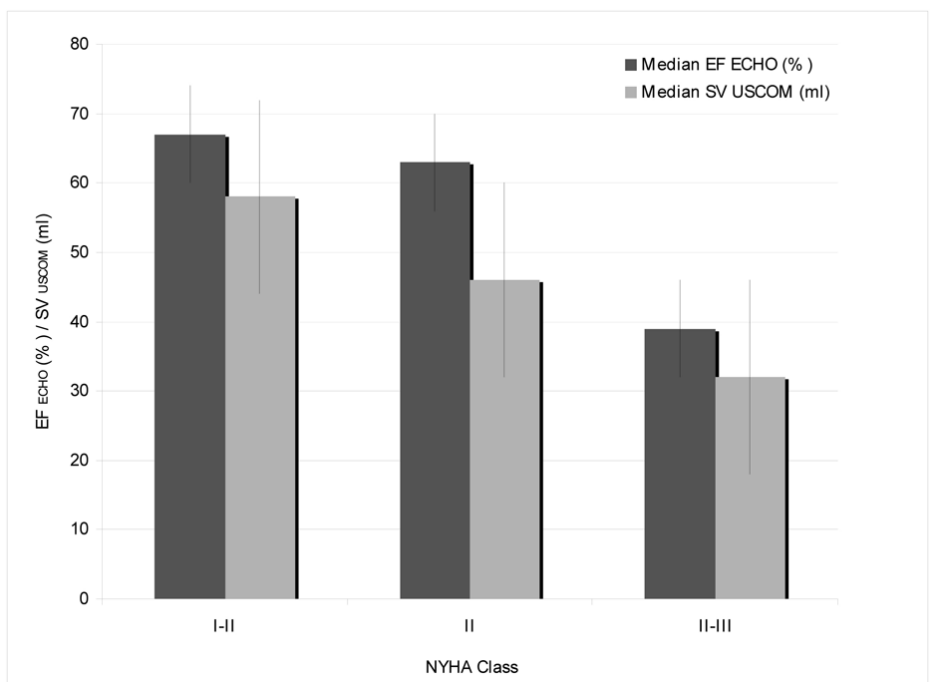
Clinical presentation by NYHA classand correlation to median EF by echocardiography and SV by USCOM.

A total of 18 of the 52 patients developed clinical signs of heart disease after receiving an anthracycline-based regimen, with 16 patient who were classified to NYHA class I-II (30.8%) and 2 patients to NYHA class II-III (3.8%). The inverse correlation of a higher NYHA class and a decrease in EF ECHO and SV USCOM is demonstrated in Figure 1.

### Cumulative anthracycline dose and cardiac function

**Figure 2:**
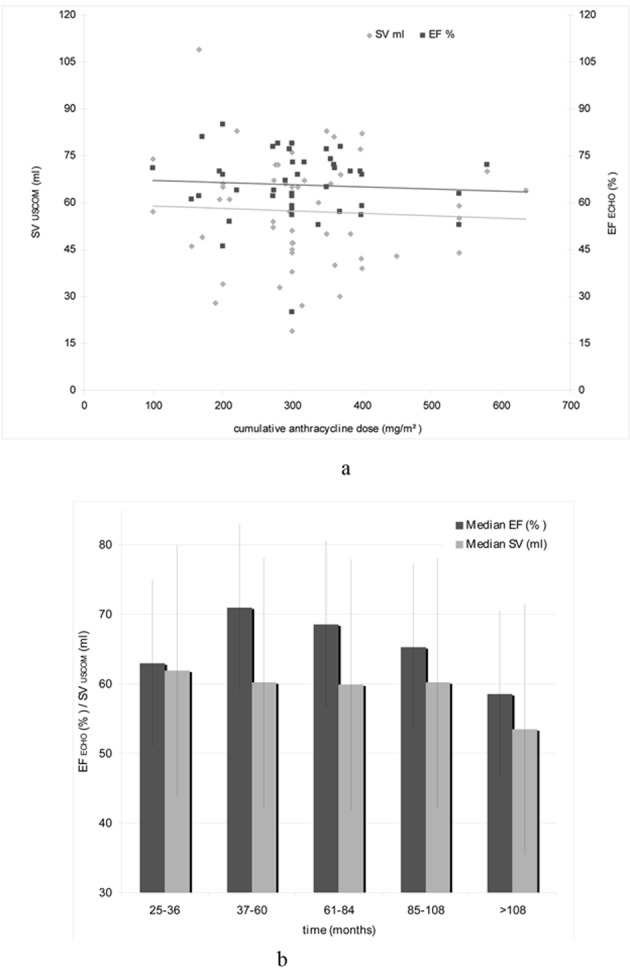
(a). Correlation of cumulative anthracycline dose (mg/m^2^), EF ECHO (%) and SV USCOM (mL). (b).Median EF ECHO (%) and SV USCOM (mL) in relation with the time interval after anthracycline-based treatment.

As the cumulative anthracycline dose is considered the most important risk factor for the development of an acute and chronic cardiotoxicity we tried to quantify late cardiotoxicity as a reduction of the stroke volume (SV) by USCOM, as well as the EF measured by echocardiography. The correlation between cumulative anthracycline dose and EF ECHO and SV USCOM is given in Figure 2a.

To investigate the relationship between anthracycline therapy and time, patients were divided into five groups depending on the elapsed time after receiving an anthracycline-based regimen and the hemodynamic investigations (25 - 36 months, 37 - 60 months, 61 - 84 months, 85 - 108 months, and > 108 months). The relationship between the time interval after treatment and EF ECHO and SV USCOM is illustrated in Figure 2b.

### hs-cTnT, NT-pro-BNP levels, and NYHA class

**Figure 3:**
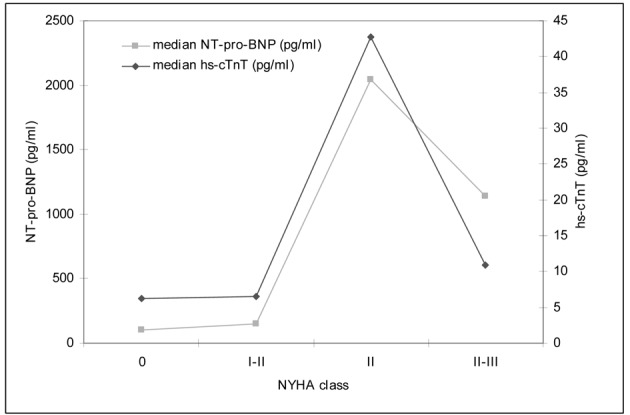
Clinical presentation by NYHA class and correlation to median cardiac markers (hs-cTnT, NT-pro-BNP).

Secondary objective of this study was the determination of myocardial markers and their correlation with the cardiac function measured as EF ECHO and SV USCOM. For all patients hs-cTnT and NT-pro-BNP was measured after anthracycline therapy at the same time of the hemodynamic investigations. A higher NYHA class was associated with an increased median level of myocardial serum biomarkers. Figure 3 shows the relationship between hs-cTnT, NT-pro-BNP and NYHA class.

## Discussion

The objectives of the present study were to assess and to quantify late cardiotoxicity after anthracycline-based chemotherapy as a reduction of EF ECHO and SV USCOM determined by echocardiography and USCOM. The intention of routine cardiac monitoring during anti-cancer treatment is the early detection of restricted cardiac function with the consequence of dose reduction or interruption (and/or application of cardioprotectants). Due to the potential risk of a late onset cardiotoxicity, the cardiac function has to be monitored years after anti-cancer treatment [34, 35].

USCOM is a noninvasive cardiac output monitor based on the transthoracic measurement of Doppler flow velocity over the aortic and pulmonary outflow tract. It is easy to operate, and hemodynamic parameters are displayed ‘beat by beat’. The technique is reported to be easily learned after a short learning period [36, 37]. Validation and reliability studies have been carried out in intensive care studies against invasive hemodynamic monitoring devices as pulmonary artery catheter and other thermodilution techniques with proven high correlation [29, 30, 38].

Knobloch and co-workers first have used CW-Doppler USCOM and NT-pro BNP measurements for monitoring the hemodynamic response in patients who received an anthracycline- or trastuzumab-based regimen for breast cancer [27, 28]. Moreover, a previous study of our group have shown, that combining real-time CW-Doppler USCOM and serum biomarkers is feasible for monitoring the immediate and chronic hemodynamic changes during an anthracycline-based regimen; the results obtained were comparable to those from echocardiography [39].

The value of serum biomarkers in patients with heart failure is well documented. Gustafsson and co-workers investigated the diagnostic and prognostic performance of NT-pro-BNP in primary care patients with suspected congestive heart failure. In summary, the mortality rate was higher in patients with NT-pro-BNP levels > 125 pg/mL than in patients with normal values (P < 0.002). This difference persisted after controlling for age, gender, and EF ECHO [20]. Knobloch et al observed an up-regulation of SV USCOM which was more pronounced in patients with high NT-pro- BNP levels (> 125 pg/mL) prior to the anthracycline infusion. They concluded that this finding might reflect a disturbed cardiac function even at baseline [27, 28].

In summary, the major findings of this study were: 1. Eighteen patients (34.6%) developed cardiac disease according to the NYHA classification. 2. We could show a trend towards a decreased EF ECHO and SV USCOM with increasing anthracycline doses in both, echocardiography and by USCOM, indicating a negative impact on myocardial function of the applied chemotherapy. 3. A higher NYHA class was associated with a markedly elevation of myocardial serum biomarkers. 4. Finally, we could demonstrate a trend towards an EF ECHO and SV USCOM decrease in relation to the elapsed time after the anthracycline-based treatment. 5. The correlation of USCOM to the ‘gold-standard’ echocardiography was evident in our trial. A decreased EF measured by echocardiography was paralleled by a corresponding reduced SV measured by USCOM.

Despite some striking observations, the results and conclusions of our study are hampered by several factors: 1). The present study was initiated as a pilot study with the aim to evaluate late cardiotoxicity with real-time CW-Doppler ultrasound cardiac output monitoring (USCOM), echocardiography and serum biomarkers after a median interval of 59 months after an anthracycline-based therapy. One can not exclude other factors that influence EF ECHO and SV USCOM as for example ischemic coronary heart disease. 2). The risk of developing cardiotoxicity increases considerably beyond a cumulative doxorubicin dose of 550 mg/m^2^ [7]. Critical cumulative anthracycline doses were not achieved in the present study. 3). The accuracy of the USCOM depends on a good flow signal. Moreover, USCOM but also echocardiography is an ultrasound-based device. Quality of measurement depends considerably from the experience of the investigator. For USCOM, it has been shown that subjects can be trained to obtain reliable SV and cardiac output estimations over the course of 20 patient assessments [36]. 4). Biomarkers as NT-pro-BNP are influenced by age, gender, renal function and co-medication with ACE inhibitors, beta blockers and diuretics. To our best knowledge, none of the included patients had received the above mentioned medications and all of them had a serum creatinine level within the normal range.

In summary, the addition of laboratory parameters to hemodynamic-based measurement gives an important contribution in terms of reliable cardiac monitoring. All findings by echocardiography were paralleled by a corresponding finding by USCOM. The increase of serum biomarkers was correlated to a higher NYHA stage, indicating a negative impact on myocardial function of the applied chemotherapy. USCOM does not replace standard methods as echocardiography in the cardiac surveillance of patients undergoing an anthracycline-based chemotherapy. But USCOM is attractive in many ways. It is easy to use, and as an ultrasound technique safe so it can be used repeatedly to measure the trend over time. Moreover, by using the USCOM device the physician will obtain a result in an unbeatable period of time. USCOM deserves closer attention in the cardiac monitoring of a cancer patients population even in those who survived their cancers. The present study justifies further clinical studies evaluating USCOM in haematology and oncology in larger, well-defined patient cohorts.
